# Recognising and Addressing Vaccine Hesitancy That Is Not Always Directly Voiced by a Child's Parents

**DOI:** 10.1111/apa.70746

**Published:** 2026-08-31

**Authors:** Alessandro Diana

**Affiliations:** ^1^ Centre for Vaccinology Geneva University Hospitals and University of Geneva Geneva Switzerland; ^2^ Unit of Development and Research in Medical Education University of Geneva Geneva Switzerland

A mother brings her nine‐month‐old child for a routine visit and everything is normal. You say ‘We will give your child the measles, mumps, rubella, chickenpox vaccine today’. The mother says nothing for 2 s and looks at the father. Then she puts the child's health record into her bag and says ‘I'd rather not do it now. We'll book something in a month or two.’

She has not refused, raised a concern or mentioned autism, aluminium or anything you have been trained to answer. What sounds like a scheduling decision is, in fact, hesitation. She is putting the vaccine off without saying so.

Every published algorithm for the vaccine conversation assumes something she has not said or done. All the guidance recommends a two‐step sequence. The first is to initiate the conversation with the parents, based on the assumption that they will agree. The second is to use empathic communication with those who resist. Motivational interviewing is the most widely adopted form of this. A clinical practice review published in 2026 [1] restated that this was about consensus and it was explicit about when the second step should begin. Recommend the vaccination, present it as the norm and go to step two if the parents express concerns or decline to have their child vaccinated. The clinical report from the American Academy of Pediatrics in 2024, from the same lead author, promoted the same approach and so has the training programme derived from it [2].

## 1 Words Are the Trigger in Every Version

Hesitancy is often spoken, but not in the words these algorithms recognise. It can be a question about timing, such as whether a vaccine can wait a few months or be given a little later. It can be a doubt about whether a child is old enough. Parents may question the recommendation itself, asking why a vaccine is advised now when their older children did not receive it. Or it can sound like a preference, such as wanting the routine schedule without one particular vaccine.

Each sounds like a technical question that the clinician is competent to answer. So they answer it correctly, with the schedule and the immunogenicity data. The questioning stops, the visit ends and nothing in the exchange registers as the parents expressing concerns or declining the vaccine. But the second step is never triggered.

The clinician has answered the question, but they have missed the hesitation behind it.

The parents have not voiced a theoretical objection. One randomised trial was built on precisely this trigger [3]. Clinicians who took part initiated the discussions with all parents, based on the presumption that they would want their child to be vaccinated and then used motivational interviewing techniques if they resisted. The authors reported no difference in under vaccination between this intervention and the normal control practices that did not explore their resistance. Motivational interviewing is well established and widely recommended for vaccine conversations [2], which makes the null result from this study more, not less, revealing. The plausible explanation is not that the method does not work, but that it was not activated in a large number of hesitant parents.

The current guidance treats offering the vaccine as the normal next step as a tool of persuasion. It is recommended because it works and, when it is presented as the norm, parents accept the recommendation more often than they debate it. Its purpose is to secure agreement, and its value is measured in uptake.

That is not wrong, but it is only part of what the recommendation does. Every recommendation also produces a response. It is easy to treat that response as noise on the way to consent, but it is information. The parent who accepts, who asks about timing, who defers, or who says nothing and glances away is telling the clinician something different, and it is something that the clinician could not have known before speaking.

The shift in approach that this perspective proposes is that the presumptive announcement is not just a recommendation. It is a diagnostic probe and its first output is the parents' response, not just agreement.

Offering the vaccine is the moment the consultation becomes readable. Acceptance, a question, a delay, a silence are all different results, and each one calls for a different next move. A firm recommendation and an open, exploring conversation are not rival philosophies or a matter of temperament. They are two stages of one process: a first step that sorts, then a second that responds. What has never been described is the step between them, namely reading the result, and that is where the hesitancy is lost.

## 2 Announce, Detect, Switch

What I propose is a different approach. It is not a fixed sequence, but a way of sorting the response (Figure 1). The clinician offers the vaccine as the normal next step, then reads what comes back. The way that the conversation progresses depends on what the parent's response reveals, not on how far along a fixed path the family has moved. A parent who quietly defers is engaged in an exploratory conversation straight away, not when they fail to be persuaded by facts that they never disputed.

**FIGURE 1:**
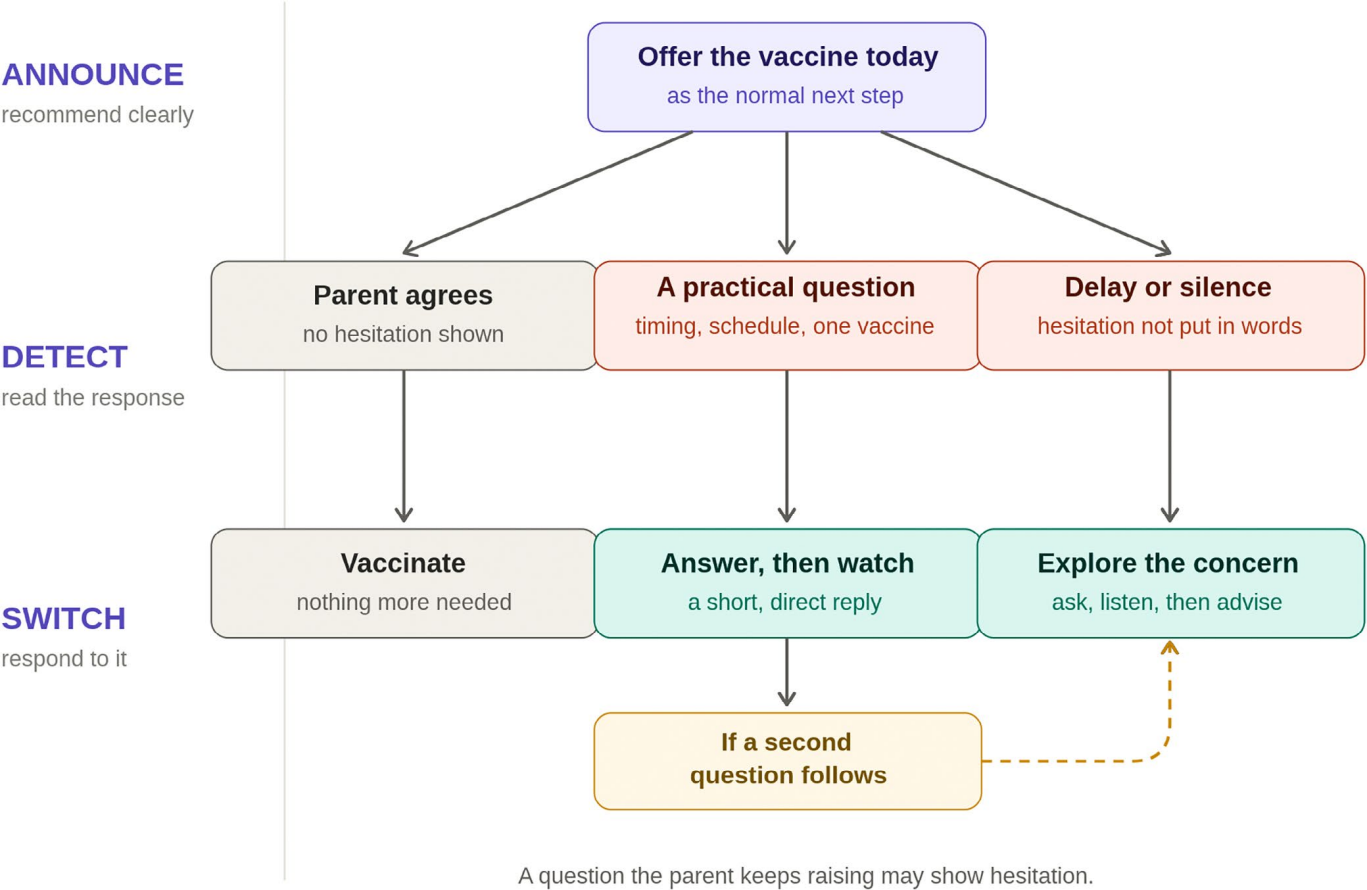
Announce, detect, switch. A clear recommendation to vaccinate is offered as the normal next step. The parents' responses are then read: agreement, a practical question or a delay or silence. The clinician responds accordingly by vaccinating, providing a short reply and watching the parents' reactions or exploring their concerns. If a second question follows the short reply, the clinician moves to exploring the concern. A question that the parents keep raising may show hesitation.

Detection has not previously been described. The signals that should prompt a change of approach are recognisable and most are spoken (Table 1). They share the same pattern: on the surface is a practical question that the clinician can answer and underneath that there is a worry that a technical answer will not resolve. Asking to rearrange the schedule tests how firm the recommendation is. Saying a child is too young questions the timing, but often ends in late vaccination. Asking why a vaccine is advised now, when older children did not receive it, challenges the recommendation itself, not its safety. Asking for most vaccines, except for one, is a refusal of that vaccine that is framed as a preference. And a vague delay or a silence is the hesitation that the parent has not put into words and only the clinician's attention can catch it.

**TABLE 1:** Five presentations of vaccine hesitancy that do not register as parents declining or expressing concerns. It covers what each sounds like, what the parents probably mean and possible ways to respond.

What the parents say	What the clinician hears	What the parents may mean	What the clinician could say
‘Could we wait until next year?’	Scheduling question	A test of how firm the recommendation is	‘We can, but is it about the timing or something about the vaccine itself?’
‘Eleven is too early for the HPV’	Judgement about the child	A deferral that often ends in late vaccination	‘Many parents feel that. What would make it feel like the right time?’
‘Why offer the varicella now, when my older children didn't have it?’	Request for information	A doubt about the authority behind the recommendation	‘That's a fair question. The advice has changed and it sounds like that shift bothers you.’
‘Can my child have the routine vaccines, but without hepatitis B.’	Preference	A targeted refusal	‘Can I ask why you feel differently about that vaccination?’
A vague deferral or silence	Nothing	An unspoken signal	‘You seem a little unsure about the vaccination. What is on your mind?’

*Note:* The words are just a suggestion and not a script.

One simple rule ties these together. A genuine request for information ends once it is answered. A question that carries hesitation, which is what motivational interviewing calls ambivalence, namely a real pull in two directions, does not. The parent accepts the answer, then raises something else. When a second question follows a perfectly good answer, the problem was never a lack of information. That is the moment to switch the conversation.

There is a catch in this approach that is worth discussion. Opening with a firm recommendation may partly work because it makes parents less likely to voice their doubts out loud. In contrast, more open questioning starts brings more doubts to the surface [4]. But that is also the difficulty, because by keeping parents quiet, by providing a firm opening statement hides the very signals the clinician needs in order to notice hesitation. This is a genuine limitation of the approach.

There is also an ethical point. Opening with a firm recommendation is only acceptable because the clinician can change course the moment a parent hesitates. Their recommendation is only justified if the clinician is also ready to notice hesitation and respond to it. A clinician who just provides a recommendation and does not notice hesitation is not being efficient. They are simply deciding on the parents' behalf.

That is primarily about training, not policy. Communication is a procedural skill, which is not acquired by reading a guideline any more than suturing is. It requires simulation and feedback. Yet the clinician's recommendation is the strongest single predictor of whether parents accept a vaccine, and communication is almost the only clinical skill for which no routine, structured training is provided. We teach the immunology of the vaccine but leave the conversation about it to improvisation.

Switzerland is a good illustration. Varicella was only added to the routine immunisation schedule in 2023, so parents routinely ask why it is recommended for their current child when their older children did not receive it. This request for information challenges the recommendation's legitimacy and is prompted by a change in policy, rather than by anything the parent has read. When recommendations actively shift, it is very common to see hesitancy disguised as reasonable questions, which can easily be mistaken for something else. It is not just clinicians that need to read these signals, as Swiss vaccinations are increasingly being delivered by pharmacists, nurses and medical assistants. Midwives also field questions, even though they do not vaccinate children. The literature focuses on physicians, but the training needs to reach all the professionals responsible for vaccinations.

What would clarify this is a future trial about the three ways of communicating. This would compare the simple recommendation to vaccinate, providing the parents with information and an empathic approach that explores the parents' concerns. Such a study would directly compare each approach with one another and not just measure whether the child was vaccinated that day. For example, it could group parents by how hesitant they are and report how often the empathic step was actually used with hesitant parents.

This proposal could, and should, be put to the test. If detection can be taught, then clinicians trained to recognise these signals should do better than those who are trained to adopt any single style of communication. If they cannot do that, then the model would fail and clinicians would simply adopt the style that tends to work best. The evidence that clinicians are poor at recognising hesitancy is a problem for the proposal, not just a footnote. A model that depends on detection is only as good as the actual detection it can produce.

There is a simpler proposition that could be used until such a trial is carried out. When a parent asks whether a vaccine could wait a month or two, it is better to treat the question as a result rather than as a request. This is one of the common presentations of vaccine hesitancy that clinicians are least equipped to recognise [5].

The sequence that follows it is short enough to incorporate into any consultation. Announce the offer to vaccinate and then read the response, including any silences, as a result rather than a reply. Only switch to a more proactive approach if the situation calls for it. This announce, detect, switch approach provides the same three moves whether the visit lasts an hour or 90 s and whether the hesitancy is spoken or, more commonly, not.

## 3 Conclusion

Most parents accept vaccination when it is clearly recommended and a short, confident recommendation is normally enough. Difficulties occur when parents hesitate but don't say why, ask to wait, question the timing or simply go quiet. Their hesitation is easy to miss, because it does not sound like a refusal. Clinicians should treat their recommendation to vaccinate as a way of finding out where the parent stands, reading their response and changing their approach if needed. The announce, detect, switch approach provides clinicians with a simple way to tackle this hesitation during the visit, before the family leaves without the vaccine. What we now need to test is whether clinicians can be taught to recognise it reliably.

## Author Contributions

**Alessandro Diana:** conceptualization, writing – original draft, writing – review and editing.

## Conflicts of Interest

The author declares no conflicts of interest.

## Data Availability

Data sharing not applicable to this article as no datasets were generated or analysed during the current study.
